# The History of Enterovirus A71 Outbreaks and Molecular Epidemiology in the Asia-Pacific Region

**DOI:** 10.1186/s12929-019-0573-2

**Published:** 2019-10-18

**Authors:** Jiratchaya Puenpa, Nasamon Wanlapakorn, Sompong Vongpunsawad, Yong Poovorawan

**Affiliations:** 1 Center of Excellence in Clinical Virology, Department of Pediatrics, Faculty of Medicine, Chulalongkorn University, Bangkok, Thailand; 2 Division of Academic Affairs, Faculty of Medicine, Chulalongkorn University, Bangkok, Thailand

**Keywords:** Enterovirus A71, Molecular epidemiology, Asia-Pacific region

## Abstract

Enterovirus A71 (EV-A71) is one of the common causative pathogens for hand foot and mouth disease (HFMD) affecting young children. HFMD outbreak can result in a substantial pediatric hospitalization and burden the healthcare services, especially in less-developed countries. Since the initial epidemic of predominantly EV-A71 in California in 1969, the high prevalence of HFMD in the Asia-pacific region and elsewhere around the world represents a significant morbidity in this age group. With the advent of rapid and accurate diagnostic tools, there has been a dramatic increase in the number of laboratory-confirmed EV-A71 infection over the past two decades. The population, cultural, and socioeconomic diversity among countries in the Asia-Pacific region all influence the transmission and morbidity associated with HFMD. This review summarizes the current state of epidemiology of EV-A71 in Asia-Pacific countries based on the most recent epidemiological data and available information on the prevalence and disease burden. This knowledge is important in guiding the prevention, control and future research on vaccine development of this highly contagious disease of significant socioeconomic implications in public health.

## Introduction

Infection by an enterovirus such as enterovirus A71 (EV-A71) can be asymptomatic or manifest as a self-limiting influenza-like illness. However, EV-A71 is one of the most important neurotropic viruses known. It is highly transmissible and infection results in hundreds of thousands of hospitalizations of children annually throughout the world, many of whom experienced severe or fatal neurologic consequences. EV-A71 has been recognized as the most common pathogen of the hand, foot, and mouth disease (HFMD), which is highly contagious and frequently affects young children below 5 years of age. EV-A71 can also occasionally cause serious neuropathology and cardiopulmonary complications, including aseptic meningitis, acute flaccid paralysis, brainstem encephalitis, and fatal myocarditis and pulmonary edema [1, 2].

EV-A71 is a member of the genus *Enterovirus* within the family *Picornaviridae*. EV-A71 belongs to the species A enterovirus, which includes 25 other serotypes [3]. Although other members of the genus can infect multiple animals, EV-A71 only infects humans. The virus has a positive-sense, single-stranded RNA genome encapsidated in a non-enveloped capsid virion. The viral genome is approximately 7500 bases in length and is flanked by 5′ and 3′ untranslated regions (UTR) and a polyadenylated tail of variable length [4]. The single open reading frame (ORF) encodes a large polyprotein, which is proteolytically cleaved by the viral protease into structural protein P1 (VP1-VP4), and nonstructural proteins P2 (2A-2C) and P3 (3A-3D).

EV-A71 is transmitted predominantly via oral-fecal route, but also through contact with virus-contaminated oral secretions, vesicular fluid, surfaces and fomites. It can also be transmitted through direct contact with patient’s aerosolized respiratory droplets [5]. EV-A71 can infect a wide range of cell types with different replicative capacity. Virus entry into susceptible host cells involves surface attachment, receptor binding and particle uptake into host cell through an endocytic pathway. The specific host cellular receptor for EV-A71 remains unknown, but until recently, there has been at least five different types of human cellular receptors identified so far. The first characterized receptor is the human scavenger receptor class B member 2 (SCARB2), also known as the lysosomal integral membrane protein II or CD36b like-2 [6]. SCARB2 was also identified to be a receptor for coxsackievirus genotypes A (CV)-A7, A14 and A16 [7]. The second characterized receptor is the human P-selectin glycoprotein ligand-1 (PSGL-1), a membrane protein expressed on leukocytes. Several studies have shown, however, that only some strains of EV-A71 utilize this receptor for cell entry [8]. The third characterized receptor is the sialic-acid-linked glycan, which is express in abundance in the respiratory and gastrointestinal epithelium cells [9]. The fourth receptor is human annexin 2 protein, which was identified as cellular host factor that interacts with EV-A71 during viral entry into human rhabdomyosarcoma (RD) cells [10]. The fifth attachment receptor is heparan sulfate glycosaminoglycan, which is widely expressed in all cell types [11]. Heparan sulfate was also observed to facilitate infection of RD cells by CV-A16, thereby serving as its receptor [12]. However a recent study identified KREMEN1 as an entry receptor for CV-A10 and other coxsackievirus A [13].

## A brief history and diagnosis of EV-A71

Historically, EV-A71 was first isolated from the feces of a female encephalitis patient in 1969 in California [14]. A retrospective analysis by a group in The Netherlands suggests that it could have emerged there as early as 1963 [15], consistent with records of probable epidemic of EV-A71 in the late nineteenth century in the United States, Europe, Australia, and Asia [16]. Between 1972 and 1990, EV-A71 outbreaks were reported in New York (1972 and 1977) [17, 18], Sweden (1973) [19], Bulgaria (1975) [20], Hungary (1978) [21], The Netherlands (1986) [22], and Brazil (1988–1990) [23]. More recently, EV-A71 and other enterovirus A infections are recognized as a major public health concern, especially after yearly HFMD outbreaks in several Asia-Pacific countries. Clinical manifestation and severity of EV-A71 and other enterovirus infections are very similar, but their genetic background and pathogenic potential are notably different. As such, early and effective diagnostic techniques are required to differentiate these enteroviruses necessary for appropriate clinical management. Virus isolation has been the traditional diagnostic method to detect EV-A71, which involves taking clinical samples from patients and culturing in a variety of cell lines of human (RD, HEK293, HEp-2, HeLa cells) or other primate origin (Vero and COS-7 cells) [24]. However, this method has gradually been replaced by more sensitive and rapid molecular diagnostics. Work pioneered by Oberste et al. [25, 26] utilizes reverse-transcription polymerase chain reaction (RT-PCR) assay to examine the VP1 region, which combined with nucleotide sequencing could reveal the viral serotypes. Nowadays, most diagnostic laboratories follow three basic techniques to definitively identify EV-A71, primarily isolation using tissue cultures, conventional immunological methods (indirect immunofluorescence and/or neutralization assay) [27], and nucleotide sequences from gene amplification using conventional and/or real-time RT-PCR [28]. The evolving methodologies used to diagnose and identify EV-A71 over the past decades have therefore confound the comparison of regional and temporal prevalence of EV-A71. In addition, variation in the detection methods used in different countries and settings may also influence the effectiveness of disease surveillance and ultimately mortality and morbidity rate reported in the literatures.

## EV-A71 circulation in the Asia-Pacific countries

EV-A71 became endemic in the Asian-Pacific region by the 1990’s and typically caused major outbreaks every 3–4 years. Countries with reported outbreaks include Malaysia [29], Taiwan [30, 31] and Singapore [32]. The overall mortality rate among patients diagnosed with EV-A71-associated HFMD in the Asian-Pacific countries has ranged from < 0.5 to 19% [33–36]. Since 1997, an unprecedented increase in the number of EV-A71 infection has primarily been attributed to the circulation of two genotypes, B and C. Here, we compile reports of EV-A71 outbreaks in different countries of the Pacific region during the last two decades.

### Australia

**Fig. 1:**
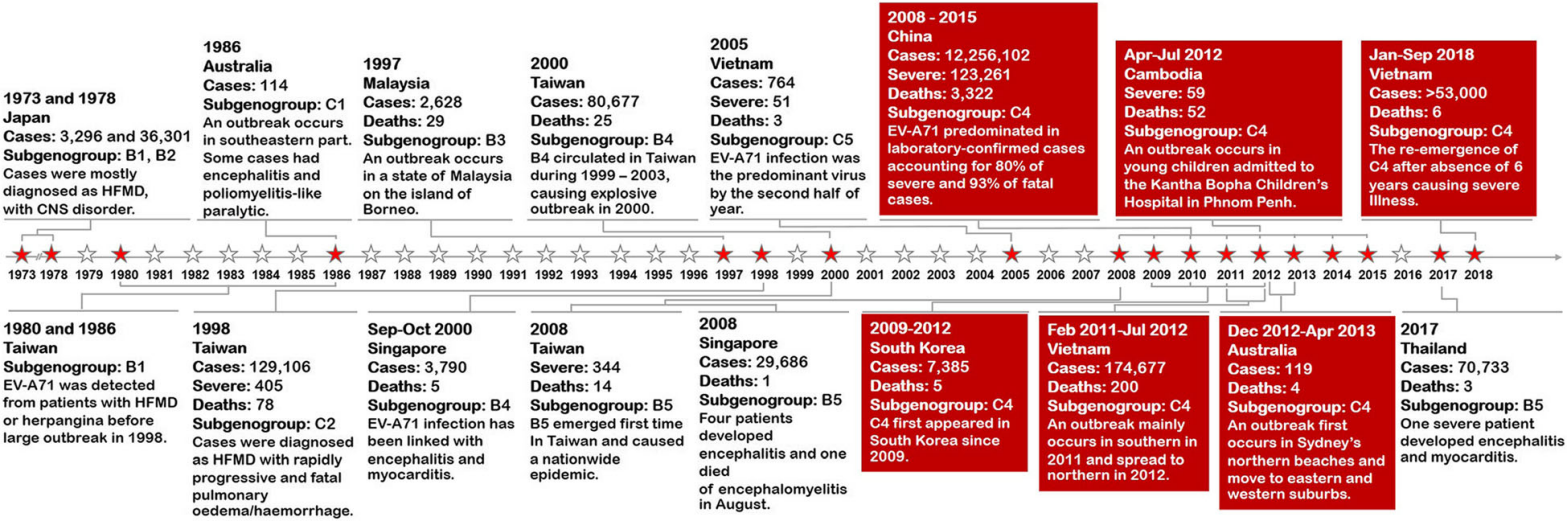
Timeline of major EV-A71 outbreaks in Asia-Pacific countries

In 1973, an outbreak was reported in Melbourne, Australia, and again in Victoria in 1986, with 114 confirmed cases of EV-A71 (Fig. 1) [37, 38]. In 1999, Australia experienced outbreak in over an 8 month period with 6000 reported cases, of which 14 were clinically severe [39]. During the summer of 2000–2001, more EV-A71 cases were reported mainly in Sydney, which resulted in approximately 200 hospitalizations and 14 severe cases [40].

An epidemic of EV-A71 infection in Sydney occurred again during the first half of 2013. This outbreak first began in Sydney’s northern beach community, then spread throughout the Sydney area [41]. An increasing number of severe neurological cases began in mid-November 2012, and HFMD cases surged in February 2013 and peaked in March. In this outbreak, there were nearly 120 severe cases reported, with EV-A71 being the predominant genotype (Fig. 1) [41, 42]. Pronounced clinical symptoms of suspected EV-A71 infection during this outbreak involved myoclonic jerks among patients with severe infection [41]. In all, there were four fatalities associated with EV-A71 neurological disease [43].

### Cambodia

Although epidemiological reports have been scarce, an unprecedented EV-A71 epidemic was noted during the first half of 2012, including 56 deaths with severe encephalitis (Fig. 1). A high case-fatality rate of EV-A71 infection (> 60%) resulted from this outbreak [44].

### China

Among the Asia-Pacific countries, China has the most number of EV-A71-associated HFMD outbreaks and the best epidemiological surveillance records in the past decade. It has been observed that the circulation of EV-A71 is particularly dynamic in this region. In 1987, an HFMD outbreak was firstly reported in Hubei province [16]. Although HFMD has been reported since the 1980’s, disease etiology was not well-recognized until 2007 when > 80,000 cases and 17 deaths were reported [45].

A comprehensive nationwide enhanced surveillance system for HFMD was established in China in May 2008 and fully implemented in July 2009 [46]. According to the Chinese Center for Disease Control and Prevention, there were approximately 13.7 million HFMD cases reported between 2008 and 2015, which include 123,261 severe cases and 3322 deaths (Fig. 1) [47, 48]. Between 2010 and 2012, the annual HFMD incidence in China during this period was at an all-time high. The prevalence ranged between 1221.3 and 1616.4 cases per million, with the most severe and fatal cases occurring in 2010 [46]. Although CV-A16 and other enteroviruses were also co-circulating, EV-A71 was observed in the majority of cases during the 8-year surveillance study (except for 2013 and 2015) [46, 48].

### Japan

EV-A71 associated with the central nervous system disorders was described in Japan in 1973 and 1978 (Fig. 1) [49, 50]. National surveillance of HFMD has since been carried out by Japan’s National Institute of Infectious Diseases in approximately 3000 pediatric medical sentinel sites by July 1981 [51]. HFMD in Japan has an epidemiological pattern with outbreaks averaging every 3 years, several of which occurred in last decade. During the summer of 2011, Japan had the largest epidemic of HFMD on record. A total of 347,362 cases were reported, with most cases occurring in children younger than 3 years of age [52]. CV-A6 infection was responsible in the majority of cases, with co-circulation of CV-A16 and EV-A71 [53, 54]. Nationwide epidemics ensued in 2013, 2015 and 2017 with 1515 cases, 1590 cases and 1900 cases, respectively [55]. The outbreak in Japan has particular relevance because of the active circulation of CV-A6 since 2011, while EV-A71 was less predominantly observed in the epidemics of 2010 and 2012 [55]. Thereafter, EV-A71 was sporadically detected from October 2014 onward, but its increase activity began at the end of 2017 when it became the predominant serotype in 2018 with approximately 70,000 reported cases [55, 56].

### Malaysia

EV-A71 infection with neurological complications and mortality was observed in Sarawak in April 1997, followed by reports in Peninsular Malaysia [57, 58]. According to the Sarawak State Department of Health, there were 2628 HFMD cases, including 29 deaths due to encephalitis and cardiac failure (Fig. 1) [59]. During this period, such encephalomyelitis associated with neurogenic pulmonary edema caused by EV-A71 infection in Kuala Lumpur were implicated in four fatal cases [60, 61]. Concurrent with adenovirus, EV-A71 involvement resulted in myocardial failure and death during the HFMD outbreak in Sibu, Sarawak [62].

The Sarawak Health Department has a sentinel surveillance program for HFMD since March 1998 [63]. It showed that large epidemics occurred almost every 3 years beginning in 2000 [63–67]. Epidemiological data suggest several common features of HFMD in Malaysia. First, the dominant EV-A71 genogroup B co-circulated with genogroup C. The monthly cases of each epidemic cycle peaked early in the year from February to April. Additionally, there was a drastic drop in EV-A71 cases in the second and third year after the epidemic cycle (2001–2002 and 2004–2005) [63]. In 2006, there were 250 reported cases with central nervous system complication, including six deaths during EV-A71 epidemics in Sarawak [65]. According to the National Public Health Laboratory, EV-A71 was the most prevalent among the endemic cases between 2008 and 2009 [68].

### Singapore

As a well-developed city-state with good public health and medical system, Singapore has long required reporting of many infectious diseases including HFMD. Reports of HFMD epidemics in Singapore have been reported in 1972 [69] and in 1981 [70]. Since then, occurrence of HFMD and aseptic meningitis associated with EV-A71 was reported in 1987 [16]. The highest recorded HFMD incidence occurred in 2000 when HFMD case numbers surged in early September, then peaked in October (3790 cases) [32]. There were a total of 76 laboratory-confirmed EV-A71 cases, of which 4 were fatal [32]. During 2001–2007, HFMD cases in Singapore were between 5187 and 20,003 cases annually [71]. Most enterovirus infections occurred in children < 4 years [71]. Monitoring of enterovirus reports showed that CV-A16 (40%) and EV-A71 (30%) predominated at several children centers, kindergartens, and schools between 2001 and 2007 [71]. In 2008, Singapore experienced its largest HFMD outbreak involving approximately 30,000 HFMD cases (Fig. 1) [72]. Throughout 2008, CV-A6 and EV-A71 were the leading types, followed by CV-A10 [72].

### South Korea

The Korea Centers for Disease Control and Prevention launched a national surveillance of enterovirus in 35 primary clinics, 105 secondary hospitals, and 40 tertiary hospitals nationwide since 1993. During 1999–2011, 4762 laboratory-confirmed enterovirus cases were recorded from the EV surveillance. Overall, around 15% of all positive samples were EV-A71, followed by echovirus 30 (13%) and CV-B5 (9%) [73]. The first reported outbreak of EV-A71 in South Korea occurred in 2000, which involved 12 hospitalization cases [73, 74]. After an absence of 6 years, EV-A71 re-emerged and became endemic in South Korea with HFMD reported every year. An upsurge in severe HFMD disease in South Korea caused by EV-A71 occurred in 2009 beginning in the spring (Fig. 1) [73]. Official records showed that there were 2427 cases, of which 94 cases of laboratory-confirmed EV-A71 infection involved complications of the CNS and 2 deaths [2]. During this epidemic, the predominance of EV-A71 also co-incited with CV-A5 and CV-A6 circulation [2].

### Taiwan

A nation of well-developed health and social infrastructure, the physician-based sentinel surveillance conducted by the Ministry of Health reported EV-A71-associated morbidity in 1980, 1981, and 1986 [30, 75, 76]. Taiwan experienced the largest epidemic in the year 1998, with an overall attack rate of 43% (Fig. 1) [30, 31, 77]. Viral transmission decreased during the summer season (July to September), and 2 epidemic waves were identified with peak incidences of 15,758 cases and 3177 cases during the week of June 7 and October 4, respectively [30, 31, 33]. In addition, unusual neurological complications were reported. In all, there were approximately 130,000 cases, of which 405 were severe and 78 were fatal [1, 30, 31].

EV-A71 reemerged in Taiwan in 2000, 2001, 2005, 2008 and 2012. More than 600 severe cases and 51 deaths were reported to the Taiwan Center for Disease Control in both 2000 and 2001 consecutive years [78]. A total of 142 cases (16 fatal) were recorded in 2005 for the entire country, and EV-A71 infection was most common in children ≤4 years of age [79, 80]. There were 373 severe confirmed cases in 2008, of which 14 were fatal (Fig. 1) [81]. Infections peaked in June with 39 confirmed cases, similar to previous EV-A71 infection in 1998 in Taiwan [81]. The marked increase in EV-A71 infection paralleled reports from the National Cheng Kung University Medical Center in southern Taiwan that same year, which documented 367 cases. This figure was much higher than the EV-A71 case number in 2007 (1 case) and 2006 (no case) [82]. Other studies also confirmed that EV-A71 was also the most prevalent genotype found in northern Taiwan among the HFMD cases in 2008 and that some affected children experienced neurological complications [83–85]. For example, two children diagnosed with HFMD presented with brainstem encephalitis and cardiopulmonary failure [83, 84]. In addition, three HFMD patients had encephalomyelitis [85]. In 2012, HFMD outbreak again reemerged in Taiwan [86, 87]. EV-A71 in Taiwan appears seasonal and often peaks in the summer [88–90].

### Thailand

**Fig. 2:**
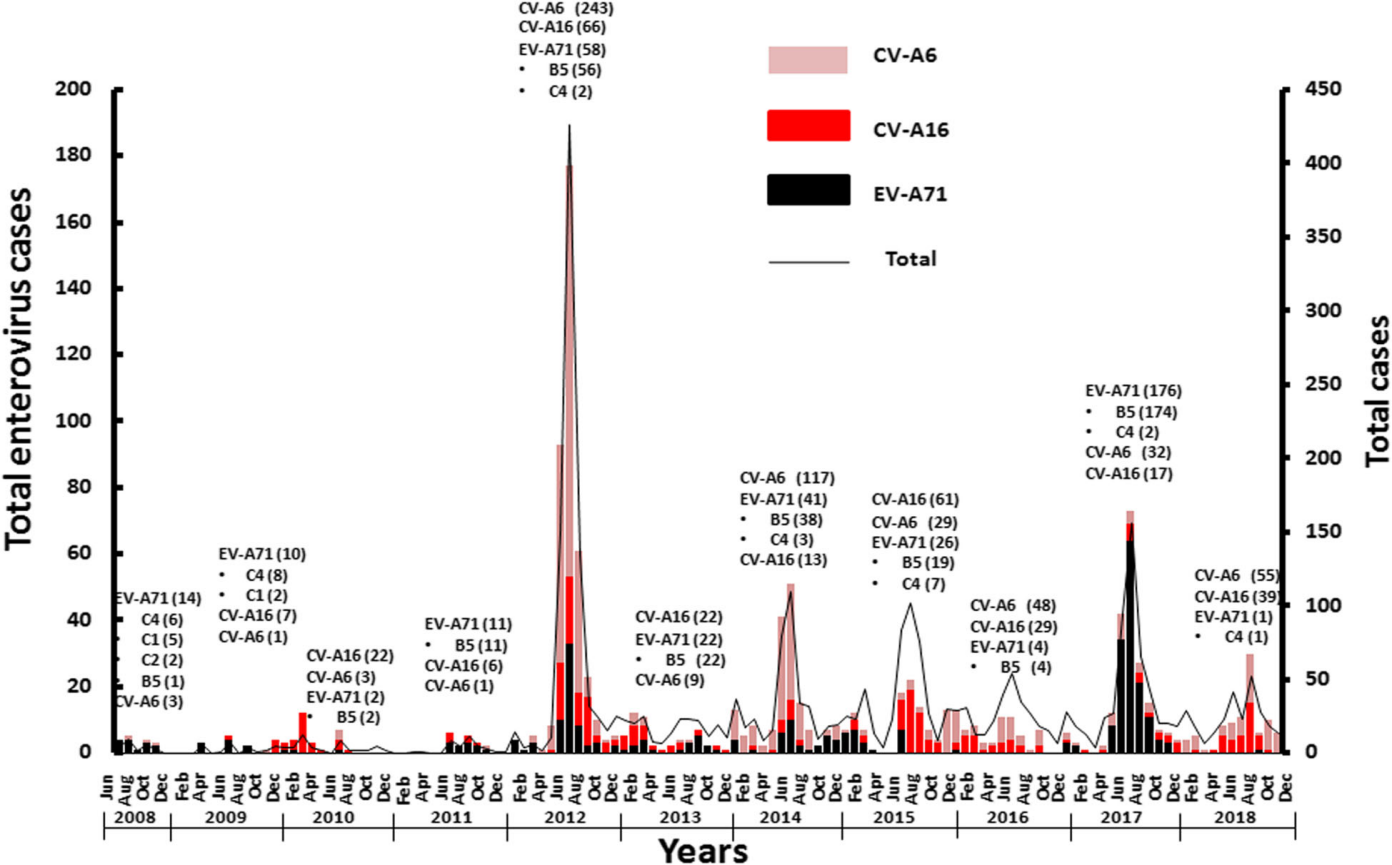
Monthly number of suspected HFMD cases and distribution of predominant enterovirus genotypes in Thailand, during 2008–2018 [92–96].

Since 2001, the Bureau of Epidemiology at the Thai Ministry of Public Health has mandated hospital-based HFMD surveillance. During 2001–2018, the ministry reported a total of 502,329 HFMD cases (ranging between 769 and 79,910 cases annually) with the highest prevalence in 2016 [91]. Reports of HFMD incidence in Thailand from 2001 to 2011 were historically low [91] and ranged between 1.2 and 28.4 cases per 100,000 population. While the number of deaths from HFMD outbreaks declined from 7 in 2006 to 2 in 2012, the number of outbreak-related cases increased from 3961 in 2006 to 45,464 in 2012 [91]. This increase was mainly due to the first large-scale HFMD outbreak in 2012, which affected primarily infants and children (Fig. 2) [92]. Most cases were associated with CV-A6, but EV-A71 infection constituted the third most prevalent type [92, 93]. Subsequently, another nationwide HFMD outbreak in 2017 was not as severe as earlier ones [94], although EV-A71 predominated in many provinces of Thailand in addition to CV-A6 and CV-A16 (Figs. 1and 2) [94].

Localized outbreaks of EV-A71 can occur regionally, as was seen in Northern provinces of Chiang Rai and Pha Yao in 2016 where the incidence rates were higher than in other regions [97]. Additionally, EV-A71 was the most prevalent genotype found in northern Thailand in which more than 55% of the cases occurred in children below 2 years of age [97]. In 2017, the Thai Ministry of Public Health had reported three mortality and an approximate count of 70,000 people who was infected with EVs [91]. The relatively rare fatal HFMD may be in part due to small proportion of enterovirus infection associated with EV-A71.

Improved tracking and surveillance has provided valuable epidemiological data in monitoring HFMD in Thailand. It is known that the percentage of outbreaks with molecular genotyping increased from 47% in 2009 to 69% in 2012 [92, 95]. Analysis of the clinical manifestation revealed that infections by coxsackieviruses and other enteroviruses differed in clinical signs and symptoms than infections caused by EV-A71 [97]. The rate of EV-A71 infection varied substantially throughout the year in Thailand and is historically highest during the rainy season [93, 96, 97]. EV-A71 infection is also detected in the drier seasons but at a lower frequency [94, 96].

### Vietnam

The first official report of EV-A71 occurred in 2003 [98]. In the second half of 2005, Vietnam experienced an HFMD outbreak caused by EV-A71 with > 700 confirmed cases, of which 51 were clinically severe and three were fatal [98]. Vietnam suffered the worst EV-A71 epidemic in its recent history with an outbreak beginning in the early of 2011 and at week 38 (September 18–24) of that year. By the end of 2012, there were almost 200,000 hospitalizations with 200 deaths, with a less than 0.5% case fatality rate (Fig. 1) [99]. Between 2013 and 2015, EV-A71 and CV-A6 were the most prevalent species among the endemic cases, followed by CV-A16 and CV-A10 [100]. In 2018, there was an increase in the number of cases, of which > 53,000 hospitalizations and 6 deaths were reported. EV-A71 was also the predominant virus in this outbreak (Fig. 1) [101].

## Molecular epidemiology of EV-A71 suggests constant viral evolution

Based on phylogenetic analysis of VP1 gene, EV-A71 is currently classified into four genotypes designated A, B, C and D [102, 103]. Genotypes B and C are individually sub-classified into B1-B5 and C1-C5. New genotypes (E, F, and G) were recently proposed, most of which were detected in central Africa, Madagascar, and India, respectively [104–106]. Genotype A was last isolated from an encephalitis case 50 years ago [14], no representative virus had been detected until 2008 [107].

Monitoring reports demonstrated that subgenogroup B1 and B2 predominated in America and Europe in the 1970s [45]. During the 80s, B2 was introduced into the United States, the Netherlands, Australia, and Japan [45]. In the early 1990s, a shift was observed in which subgenogroup C1 replaced B2 as the predominant genotype [45]. In contrast, subgenogroup B3 appears to be extinct since representatives of genotype in Singapore has not been found since 1999. Between 2000 and 2010, C2 was the predominant subgenogroup observed in AFP surveillance in Philippines [108]. Subgenogroup C3 was only isolated in South Korea in 2000 with sporadic cases (Table 1) [109].

**Table 1:** EV-A71 subgenogroups detected in Asia-Pacific Region, 1997–2018 [30, 36, 39, 41, 44, 45, 59, 67, 76, 82, 86, 87, 94–96, 98–101, 109–116].

	1997	1998	1999	2000	2001	2002	2003	2004	2005	2006	2007	2008	2009	2010	2011	2012	2013	2014	2015	2016	2017	2018
Australia	B3	B3, C2	B3, **C2**	**B4**, C1	B4		C1	C1									C4					
Brunei										B5												
Cambodia																C4						
China		C4		C4	C4	C4	C4	C4			C4	A, C4	C4	C4	C4	C4	C4	C4	C4			
India					D	D	D					D, G		D	C1, D,	C1, D						
															G							
Indonesia																				B5		
Japan	B3, B4,	C2	C2	**B4**, C2	C2	B4, C2,	B5, C4	C4		C4	C4	C2	C2	C2								
	**C2**					C4																
Laos															C4							
Malaysia	**B3**, B4,	C1	B4, B5	**B4**, B5	B4, C1	C1	**B5**, C1		B5, C1	B5	B5	B5	B5	B5	B5	B5						
	C1, C2		C1	C1																		
Mongolia											C4			C4								
Philippines				C2		C2			C2					C2								
Singapore	B3, B4	B3, C1	B3	B4	B4	B4, C1				B5		B5										
South				C3			C4				C4	C4	C1, **C4**,	C4	C4	C4						
Korea													C5									
Taiwan		B4, **C2**,	B4	B4	B4	B4, C4	B4, B5	C4	C4	C5	B5, C5	B5	B5	C4	B5, C4	**B5**						
		C4																				
Thailand					B4, C1	C1	C1	C1		B5, C1,	B5, C1, C2,	B5, C1, C2,	B4, B5,	B5	B5, C4	**B5**, C4	**B5**, C4	**B5**, C4	**B5**, C4	**B5**, C4	**B5**, C4	
										C4, C5	C4, C5	C4, C5	C1, C4									
Vietnam									C1, C4,						C4	C4	B5					**C4**, B5
									**C5**													

Bold indicates predominant genotype

**Fig. 3:**
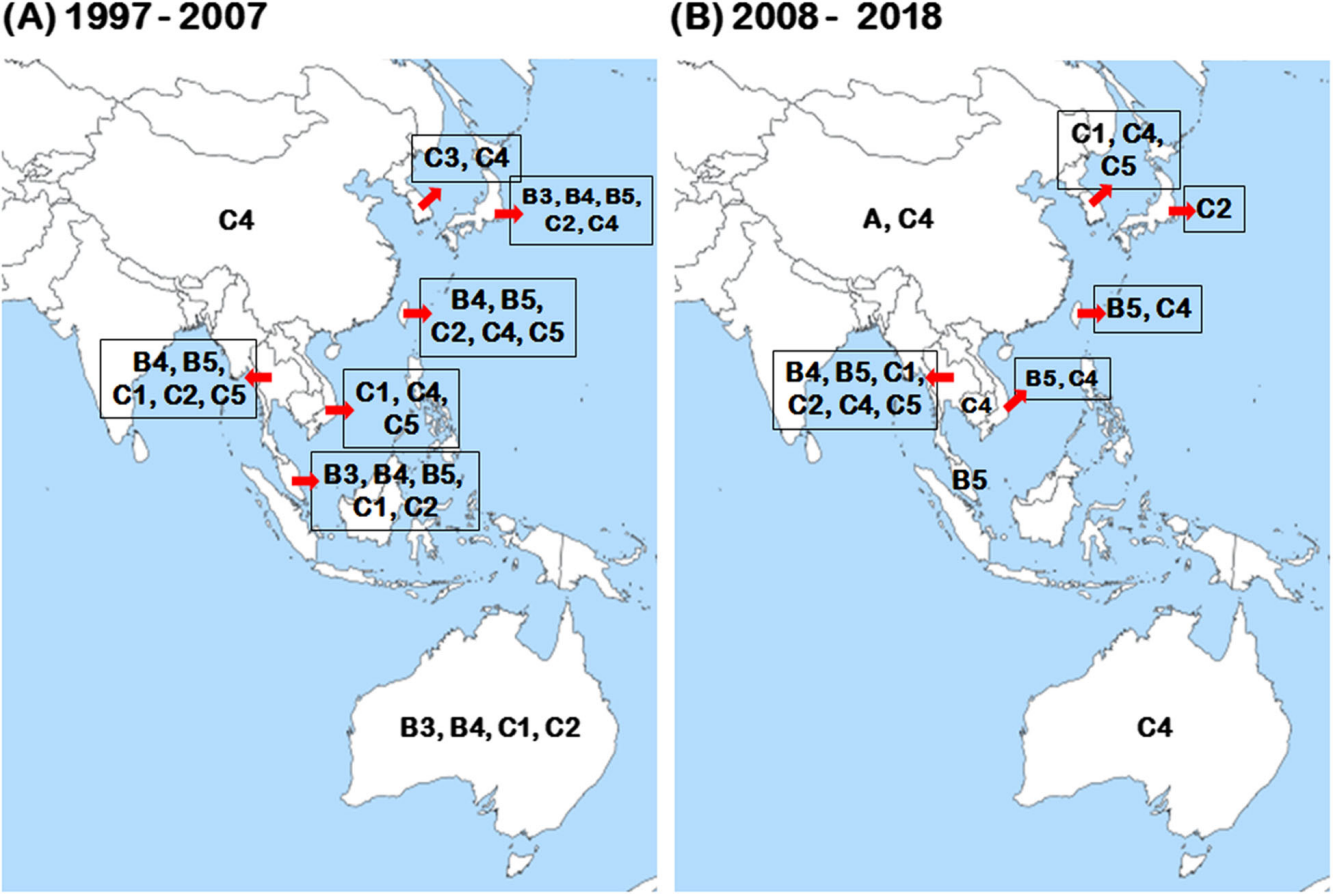
Distribution of EV-A71 subgenogroups during (**a**) 1997–2007 and (**b**) 2008–2018

In the first major HFMD outbreak in the Asia-Pacific region reported in Malaysia in 1997, molecular characterization showed that it was related to subgenogroups B3, B4, C1, and C2 (Fig. 3). Specifically, the predominance of B3 was specifically associated with fatal cases [59]. Later on in 2000 and 2003, B4 and B5 were the predominant subgenogroups (Fig. 3) [45]. Infections caused by subgenogroup C1 cases have generally been sporadic in Malaysia between 1997 and 2005 (Table 1) [45]. Subgenogroup B5 was detected in Brunei (2006) [117] and Indonesia (2016) [118]. Moreover, subgenogroup D, G and C1 circulated in India as endemic viruses between 2001 and 2012 [106].

In 1998, subgenogroup C4 emerged for the first time in the western Pacific region and caused severe epidemic beginning in China in 2008 [36, 110, 111]. C4 subsequently expanded to the rest of the region and caused major epidemics in several countries. Some of these outbreaks were associated with severe neurological complications and fatality cases, such as in Australia (2013) [41], Cambodia (2012) [44], Laos (2011) [119], Mongolia (2007, 2010) [120, 121], South Korea (2009–2012) [112], Vietnam (2011–2012 and 2018) (Table 1 and Fig. 3) [99, 101].

In Australia, outbreaks associated with subgenogroup C1 were reported in Victoria in 1986 and subgenogroup C2 in Western Australia in 1999 [39]. A subgenogroup B4 epidemic was also reported in Sydney in 2000–2001 [39]. A switch from subgenogroup B4 to C4 in early 2013 was associated with a severe outbreak in Sydney (Table 1 and Fig. 3) [41].

In Taiwan, the yearly changes in EV-A71 subgenogroup distribution reflect the typical dynamic of enterovirus strain co-circulations in a given region. For example, although subgenogroup B1 circulated in 1980 and 1986 [30, 75, 76], the increase in HFMD incidence in Taiwan in 1998 strongly associated with subgenogroup C2, eclipsing B1 as causative subgenogroup [30, 113]. In 2000 and 2001, the endemic dominant subgenogroup in Taiwan is B4 [76, 113], which changed to C4 after 2004 [76]. The emergence of the novel subgenogroups B5 has resulted in a large-scale nationwide epidemics in 2008 and 2012 (Table 1 and Fig. 3) [82, 86, 87, 114].

In Thailand, the distribution of EV-A71 subgenogroups varied by year [115]. Since 2001, enteroviruses monitoring in Thailand indicated the circulation of subgenogroups B4, B5, C1, C2, C4, and C5 (Fig. 3) [95, 115, 116]. During 2001 to 2004, molecularly confirmed EV-A71 belonged to subgenogroup C1, with subgenogroup B4 detected only rarely. However, notably, compared with the other subgenogroups, B5 first appeared in Thailand in 2006 and became the predominant subgenogroup beginning in 2010 (Table 1 and Fig. 3) [96]. During the EV-A71 B5 outbreak in 2017, two patients with subgenogroup C4 were also detected in the northern part, indicating the co-circulation of B5 and C4 in Thailand [94]. Subsequent subgenotypic replacement events of EV-A71 in Thailand have been observed in a different context. For instance, the prevalence of subgenogroup C4b displaced C4a in 2008 [115]. These observations provide evidence for the role of natural selection pressure in the replacement of the latter subgenogroups [122]. The existence of recombination within genome regions of subgenogroup B5 and C4 in Thailand was also demonstrated through comparison the results of similarity plot and bootscan analyses [123, 124].

In Vietnam, the co-circulation of C1, C4, and C5 subgenogroups responsible for HFMD outbreak in 2005 suggested the predominance of C5 in most affected children [98]. The emergence of subgenogroup C4 was implicated in a large-scale nationwide epidemic between 2011 and 2012 [99]. Subsequent emergence of subgenogroup B5 in 2012 became the dominant subgenogroup in 2013 [100]. The nationwide outbreak in Vietnam, which began in 2018, occurred after the absence of subgenogroup C4 for 6 years (Table 1 and Fig. 3) [101].

## Concluding Perspectives

An upsurge in HFMD outbreaks associated with EV-A71 occurred during the last decade. The changing epidemiology of EV-A71 in Asia-Pacific countries has seen patterns of recurring outbreaks every 2–3 years with varying frequency and clinical severity. Outbreaks in Asia have shaped the development of fast and reliable multiplex real-time RT-PCR specific towards the most prevalent viruses associated with HFMD, namely EV-A71, CV-A6, and CV-A16. As other EV types emerge, which could potentially replace the current viral circulation, new methods may be required to identify them with increasing accuracy. We have already seen that outbreaks in mainland China cause predominantly by the subgenotype C4 resulted in its inclusion in the vaccine. With new EV-A71 outbreaks, researchers gained additional genetic sequence information and clinicians identify additional range of symptoms, which assist in establishing patterns in disease progression and clinical outcome so valuable in the management of symptoms. Increased awareness of EV-A71 infection in the communities can potentially stem widespread transmission seen in past outbreaks, and fatalities associated with EV-A71 outbreaks appear to be on the decline, at least for now.

Currently, the availability of EV-A71 vaccine approved for use in some Asian countries may offer a partial solution in blunting disease transmission, but the lack of convincing evidence for the induction of cross-protection among the diversity of subgenogroups in circulation throughout the region and globally, including CV-A6 and CV-A16 associated with HFMD, remains to be addressed. In addition, it remains unclear what the optimal target age group and schedule for vaccination should be. Furthermore, the potential of the widespread use of the vaccine affecting the landscape of other EV-A71 subgenotypes in circulation remains another concern. Ongoing development of multivalent vaccines demonstrating cross-protection against EV-A71/CV-A6/CV-A16 could provide additional benefits towards the reduction of HFMD outbreaks. Additional strategies in preventing EV-related diseases burden, including the establishment of a regional HFMD disease network, transnational co-operation in vaccine research and evaluation, and standardized diagnostic methodologies with defined clinical characterization on a disease severity scale may assist in a more accurate capture of disease impact in this highly dynamic and culturally diverse region.

## Data Availability

Not applicable.

## Abbreviations

CV-A: Coxsackievirus A
EV-A: Enterovirus A
EV-A71: Enterovirus A71
HFMD: Hand foot and mouth disease
ORF: Open reading frame
PSGL-1: Human P-selectin glycoprotein ligand-1
RD: Rhabdomyosarcoma
RT-PCR: Reverse transcription polymerase chain reaction
SCARB2: Human scavenger receptor class B member 2
UTR: Untranslated regions
